# Implications of *AlphaFold*2 for crystallographic phasing by molecular replacement

**DOI:** 10.1107/S2059798321012122

**Published:** 2022-01-01

**Authors:** Airlie J. McCoy, Massimo D. Sammito, Randy J. Read

**Affiliations:** a Department of Haematology, Cambridge Institute for Medical Research, University of Cambridge, Hills Road, Cambridge CB2 0XY, United Kingdom

**Keywords:** protein structure prediction, crystallographic phase problem, molecular replacement, *AlphaFold*2

## Abstract

The *AlphaFold*2 results in the 14th edition of Critical Assessment of Structure Prediction (CASP14) showed that accurate (low root-mean-square deviation) *in silico* models of protein structure domains are on the horizon, whether or not the protein is related to known structures through high-coverage sequence similarity. As highly accurate models become available, generated by harnessing the power of correlated mutations and deep learning, one of the aspects of structural biology to be impacted will be methods of phasing in crystallography. Here, the data from CASP14 are used to explore the prospects for changes in phasing methods, and in particular to explore the prospects for molecular-replacement phasing using *in silico* models.

## Abstract 2

The implications of the *AlphaFold*2 protein structure-modelling software for crystallographic phasing strategies are discussed.

## 1. Introduction

**Figure 1:**
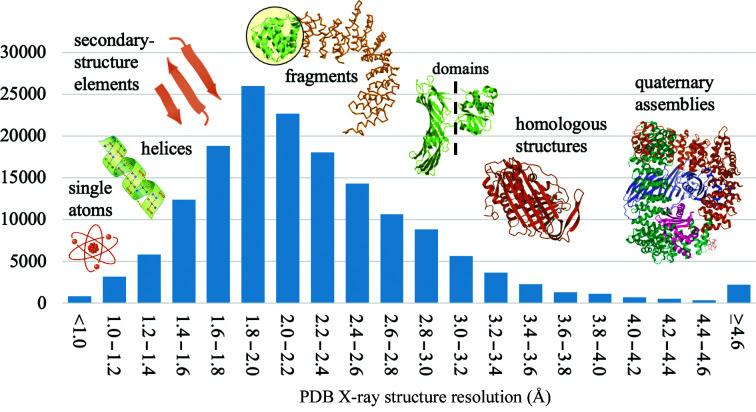
Histogram of the distribution of structures in the PDB by resolution. The relationship between the resolution of the data and the size of the models that are appropriate for molecular replacement is indicated.

The quality of a model for phasing a crystal structure by molecular replacement, for a given diffraction resolution limit, depends on the root-mean-square deviation (r.m.s.d.) of the model to the target structure and the fraction of the total scattering (*f_m_*) that it represents. Broadly, as the resolution of the experimental data decreases, the *f_m_* must increase (Fig. 1 ▸). Specific predictions based on r.m.s.d., *f_m_* and data resolution guide molecular-replacement phasing strategies in individual cases (Oeffner *et al.*, 2018 ▸).

When the data resolution extends to better than ∼1 Å, the model can be as tiny as a single atom. A single atom can be considered to be the perfect *in silico* substructure model, with an r.m.s.d. of zero to the target structure, although overall the structure factors calculated from this model have large errors because of the extremely low *f_m_*. Following single-atom molecular replacement, log-likelihood gradient completion can rapidly locate the remaining ordered atoms and for these resolutions the phase problem is considered to be solved (McCoy *et al.*, 2017 ▸).

Small secondary-structure elements of helix or β-sheet are viable models when the data extend to better than ∼2.2 Å resolution, and in some cases this technique extends to resolutions of 2.5 Å. The atomic coordinates of the model can be extracted from known structures or generated *in silico* (Glykos & Kokkinidis, 2001 ▸). Success does not necessarily require a homologous model with a sequence identity of over 30%, despite this being a commonly quoted metric for the success of molecular replacement (Scapin, 2013 ▸). Density modification and model building, which are significantly more powerful at higher resolutions, are central to structure completion with this method. Accurate fragments are regularly used for molecular replacement in software such as *ARCIMBOLDO* (Sammito *et al.*, 2014 ▸, 2015 ▸; Rodríguez *et al.*, 2009 ▸) and *AMPLE* (Bibby *et al.*, 2012 ▸; Rigden *et al.*, 2018 ▸; Simpkin *et al.*, 2019 ▸). For these resolutions, the phase problem is also largely considered to be solved. The median resolution in the PDB is 2.2 Å, making this approach possible for many present-day crystallographic structures. Supplementary Fig. S1 illustrates the theoretical reasons to expect highly accurate fragments to be easy to place by molecular replacement at these resolutions.

When the experimental data extend to lower than ∼2.2 Å resolution the models required for molecular replacement must represent, to at least some degree, the fold of the target protein (the hydrophobic core or more). A high *f_m_* and a low r.m.s.d. become progressively more important as the resolution decreases, and by ∼3.0 Å resolution, in a typical crystal, a whole-structure model with less than 1 Å r.m.s.d. would be required for successful molecular replacement and model completion. This is the zone (sub-3.0 Å) in which homologs, template-based modelling and *in silico* models become particularly valuable.

For those targets distantly related to a homologous structure, early attempts at template-based modelling (as catalogued by the Critical Assessment of Structure Prediction, CASP) generally increased (rather than decreased, as was the aim) the r.m.s.d. to the target. CASP7 was the first CASP to show that template-based modelling improved models for molecular replacement. From this CASP also came the first case in which an *in silico* structure prediction for a natural protein with an asymmetric, globular fold was successfully used for molecular replacement, albeit retrospectively (Qian *et al.*, 2007 ▸). Since then, an *in silico* model has been used in the real-world molecular-replacement phasing of the peptido­glycan polymerase RodA (Sjodt *et al.*, 2018 ▸).

Since CASP7, CASP has included a metric for scoring individual model predictions based on their usefulness in molecular replacement, with steady progress in each challenge round. From CASP13 it was demonstrated that not only accurate coordinates, but also accurate estimates of the errors in the coordinates, were critical for successful molecular replacement (Croll *et al.*, 2019 ▸).

There are several pipelines for molecular replacement with *in silico* models. One of the first was *CaspR*, which made use of models produced by *MODELLER* (Claude *et al.*, 2004 ▸). The first iteration of the *AMPLE* pipeline developed a cluster-and-truncate approach to the use of rapidly computed *ab initio* models generated by *Rosetta* or *QUARK* (Bibby *et al.*, 2012 ▸). In further developments, *AMPLE* has been extended to use structure predictions from the GREMLIN and PconsFam databases (Simpkin *et al.*, 2019 ▸). Models from *I-TASSER*, generated by full-length iterative structural fragment re­assembly, have been incorporated in the *I-TASSER-MR* server, which uses progressive sequence truncation to edit the models for molecular replacement (Wang *et al.*, 2017 ▸). *AWSEM-Suite* combines both homology model templates and coevolutionary information with the physico-chemical energy terms of *AWSEM* (Jin *et al.*, 2020 ▸). In our own collaborations, the *phenix.mr_rosetta* pipeline (Terwilliger *et al.*, 2012 ▸) can use *Rosetta* to rebuild template structures prior to attempting molecular replacement. We also use *Rosetta*, extended to include a term for fit to the electron density (DiMaio, 2013 ▸), to rank putative molecular-replacement solutions and to rebuild very poor models after molecular replacement.

## 2. CASP14

CASP14 has established a leap in protein structure prediction. The primary CASP metric for ranking models and modelling groups is the GDT_TS (Global Distance Test Total Score), a structure-similarity measure designed and developed for the structure-alignment program *LGA* (*Local–Global Alignment*) as an alternative to the r.m.s.d. (Zemla, 2003 ▸). The GDT measures the percentage of C^α^ atoms that are found within certain distance cutoffs of one another between the model and target (either dependent upon or independent of a sequence alignment): the cutoff distance(s) must be defined for each reported GDT value. The GDT_TS is the average of four cutoff distances (1, 2, 4 and 8 Å). Higher GDT values are achieved with better models, in contrast to the r.m.s.d., where lower values are better. In circumstances where a score that is sensitive to smaller differences is needed, the GTD_HA is used; this is a variant of the GDT_TS in which the cutoff distances are 0.5, 1, 2 and 4 Å.

Harnessing the power of correlated mutations, contact predictions and deep learning, the AlphaFold2 group (group 427 in the CASP14 numbering) from the commercial organization DeepMind (Service, 2018 ▸; Callaway, 2020 ▸) were ranked first on *Z*-score for GDT_TS, reaching values over twice, and up to three times, those of the second and subsequent ranked groups, depending on the classification of targets considered. This was achieved on a background of major improvements from other groups, including the Baker group (BAKER group 473, ranked second, and BAKER-experimental group 403, ranked third; Hiranuma *et al.*, 2021 ▸), who used similar methods in academic settings.

For the first time, structures submitted to CASP as targets were phased with the help of models provided during the assessment (Kryshtafovych *et al.*, 2021 ▸). For target T1058, the structure was solved by MR-SAD with the *AlphaFold*2 models after attempting molecular replacement with homologous structures, domains thereof and server models. For T1089, the *AlphaFold*2 models gave a far higher molecular-replacement signal than using trimmed ensemble models. For T1100, several of the models submitted to CASP, including the *AlphaFold*2 models, gave a molecular-replacement solution where the NMR structures of individual domains failed. The *AlphaFold*2 model for T1064 has also been used to solve the SARS-CoV-2 ORF8 structure retrospectively by molecular replacement (Flower & Hurley, 2021 ▸).

## 3. *AlphaFold* and *RoseTTAFold*

While this paper was being reviewed and revised, two major developments occurred that have made models of the quality of those submitted to CASP14 readily available to all. Firstly, the *AlphaFold*2 algorithms have been published (Jumper *et al.*, 2021 ▸), together with release of the source code, enabling the prediction of any protein structure of interest. In addition, predicted structures have been made available in a database hosted at the European Bioinformatics Institute for all proteins from a substantial number of proteomes; further releases are promised (Tunyasuvunakool *et al.*, 2021 ▸). Secondly, *RoseTTAFold* has been developed and published by the Baker group (Baek *et al.*, 2021 ▸); it has been made available as part of the *Robetta* server, and the source code has also been released. *RoseTTAFold* is a deep-learning algorithm with features inspired by what was revealed about *AlphaFold*2 at CASP14.

## 4. Molecular-replacement assessment

Suitability for molecular replacement is one of the metrics for high-accuracy assessment in CASP, including CASP14 (Pereira *et al.*, 2021 ▸; Millán *et al.*, 2021 ▸). The assessment uses a log-likelihood gain (LLG) calculated in *Phaser* (McCoy *et al.*, 2007 ▸; Read & McCoy, 2016 ▸). The likelihood is the probability that the data would have been measured given the model, and the log-likelihood gain is the difference between the log-likelihood of the model and that calculated from a random distribution of the same atoms (Wilson, 1949 ▸).

An important component of the scoring of models with the LLG is the incorporation of the estimated error in coordinates. As part of the modelling, groups are encouraged to estimate the error (Δ) in each atomic position and to record this estimate in the *B*-factor column of the deposited PDB file. This error estimate can be converted to a *B* factor for each atomic position through the relationship *B* = 8π^2^Δ^2^/3 and thereby used to weight each atom in the LLG calculation appropriately. Accurate estimates of the error improve the LLG (Bunkóczi *et al.*, 2015 ▸; Croll *et al.*, 2019 ▸), and in practice will add value to the models by increasing the signal in molecular-replacement searches.

If, during molecular-replacement searches, a pose of a model has an LLG over a certain space-group-dependent value (60 in nonpolar space groups, 50 in polar space groups and 30 in *P*1), the pose is probably correct. However, achieving this LLG is not sufficient to determine whether the full structure can be traced and refined to a point suitable for interpretation, publication and deposition; this also depends on the accuracy and completeness of the model and the resolution of the data. Testing the ability of a model to phase includes validation of these steps downstream of nominally successful model replacement.

### 4.1. Model parameters

Before structure solution, the LLG that will be achieved for a correct pose of a model can be estimated using the ‘expected LLG’ (eLLG; McCoy *et al.*, 2017 ▸). We have previously shown that the eLLG for each reflection *hkl* can be calculated from the σ_A_ parameter (Read, 1986 ▸):

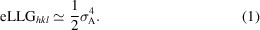

The total eLLG is the sum of this value over all reflections *hkl*. The resolution-dependent σ_A_ term is approximated with a four-parameter curve which includes the r.m.s.d., the *f_m_* and also two solvent parameters which affect the σ_A_ value at resolutions lower than approximately 8 Å (Murshudov *et al.*, 1997 ▸). For reflections higher than 8 Å resolution, this curve is dominated by the dependence on the square root of *f_m_* and an exponential dependence on r.m.s.d.^2^. For each reflection *hkl* (resolution *d_hkl_*),

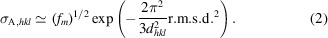

**Figure 2:**
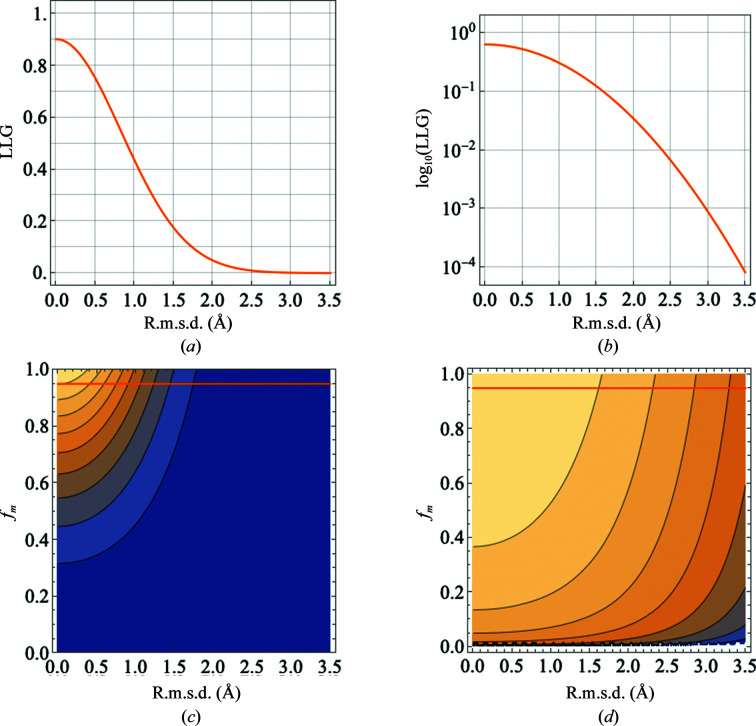
Illustration of the relationship between r.m.s.d., *f_m_* and LLG per reflection (equation 2). (*a*) LLG for *f_m_* = 0.8, linear scale. (*b*) LLG for *f_m_* = 0.8, logarithmic scale. (*c*) Contour plot showing LLG for r.m.s.d. versus *f_m_*, linear scale. (*d*) Contour plot showing LLG for r.m.s.d. versus *f_m_*, logarithmic scale. The value *f_m_* = 0.8 is shown with an orange line on the contour plots.

Since the eLLG is a good estimate of the LLG when there are no pathologies in the data (Oeffner *et al.*, 2018 ▸), the above equation also shows the relationship between r.m.s.d., *f_m_* and the LLG. The dependence of the LLG on r.m.s.d. and *f_m_* is shown in Fig. 2 ▸.

Any parameter that correlates strongly with r.m.s.d. (such as the GDT_HA, when the GDT_HA is high) will show the same relationship to the LLG.

The r.m.s.d. is only useful for predicting the LLG when the same r.m.s.d. value describes the difference between the model and target both locally and globally. If it varies between regions, the r.m.s.d. is dominated by the regions where the r.m.s.d. is large, whereas the LLG score will be dominated by the regions where the r.m.s.d. is low. In practice, there will be regions that are better modelled than others, and the LLG obtained from a model will be higher when the estimated errors in each coordinate are good and are incorporated into the electron-density calculation via the *B* factor, as described above.

### 4.2. Targets

There were 33 crystal structures provided as modelling targets for CASP14. Of these, 31 have a single protein sequence (sometimes in multiple copies) in the asymmetric unit and two have two protein sequences. The relationship between the naming of the targets and the crystal structures is not straightforward. In 30 of the 31 cases with a single sequence there is a one-to-one correspondence between a sequence and a CASP target number (for example T1032). The exception is the case of the structure with PDB code 6vr4, where there are nine separate targets (T1031, T1033, T1035, T1037, T1039, T1040, T1041, T1042 and T1043), each representing between 95 and 404 residues of the full polypeptide sequence of 2194 residues, two copies of which are present in the asymmetric unit. In 11 of the 30 cases the full sequence is deemed to be ‘multidom’ (multi-domain) and is also divided into two, three or four domains; these are treated as additional, separate targets, with the suffix ‘-D1’, ‘-D2’, ‘-D3’ or ‘-D4’ added to the target number for the whole structure (for example, T1024 is divided into T1024-D1 and T1024-D2). In eight of the 11 ‘multidom’ targets the whole sequence target is referenced suffixed with ‘-D0’ (for example T1038-D0) and not simply by the target number alone, as in the other three of the 11 targets (for example T1024). The 19 of the 30 cases that are not ‘multidom’ have a single domain defined within the full sequence, and the target is given the suffix ‘-D1’ (for example T1032-D1). In the two of the 33 cases where there are two sequences in the asymmetric unit, the corresponding two targets are named with the same target number with the suffix ‘s1’ or ‘s2’ added (T1046s1 and T1046s2; T1065s1 and T1065s2). Neither of these divide their constituents into domains, and the single targets are given the ‘-D1’ suffix.

We also considered one other target, PDB entry 6un9, corresponding to target T1048, which was cancelled from CASP14 because of a lack of tertiary structure; it is a single sequence that folds into a single helix and forms a coiled coil. A model for this structure was also prepared by the *AlphaFold*2 group before it was cancelled.

In total, we considered 72 CASP14 targets from the 34 crystal structures when the domains of ‘multidom’ targets are included in the total (Table 1 ▸). Crystallographic details of relevance to the difficulty of structure determination, such as the resolution limit and the number of copies in the asymmetric unit, are discussed below in the context of the molecular-replacement trials.

### 4.3. Classification of targets

**Figure 3:**
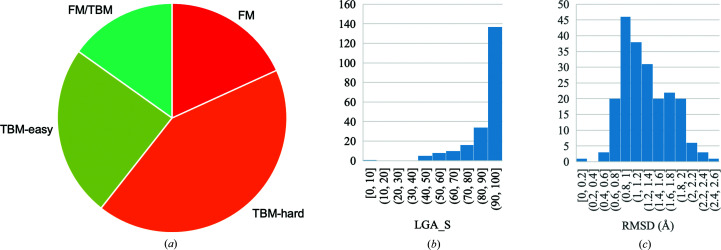
Classifications and accuracy for the 34 crystal structures in CASP14. (*a*) Proportion of the different modelling categories FM (free modelling) and TBM (template-based modelling). PDB entry 6vr4 was counted as a single FM target. Histogram of distribution of all five submitted *AlphaFold*2 models for 44 crystallographic targets of interest for (*b*) LGA_S and (*c*) RMSD.

CASP classifies the targets by modelling difficulty in four categories: free modelling (FM), template-based modelling (TBM-easy and TBM-hard) and structures on the boundary between free modelling and template-based modelling (FM/TBM). In the set of crystal structures, there was a good representation of all four classes (Fig. 3 ▸ *a*). All but two of the structures were from lower organisms (viruses, bacteria, archaebacteria and tetrahymena), and these two structures were classed as TBM, which reflects the high coverage of fold space that has now been achieved in higher organisms (Supplementary Table S1).

### 4.4. Metrics for target quality

For the purposes of judging models for molecular replacement, the model r.m.s.d. and *f_m_* are the important metrics.

Of the metrics reported by CASP, the sequence-independent *LGA* (4 Å) parameters RMSD and LGA_S are most closely allied with the r.m.s.d. and *f_m_*. The RMSD is the root-mean-square deviation for the subset of C^α^ atoms from the model that correspond to the residues from the target structure in the sequence-independent *LGA* superposition. LGA_S is the sequence-similarity score, which is a combination of the GDT score and the LCS score, where the LCS is the longest continuous segment (as a percentage of the total sequence) that can fitted under an r.m.s.d. of a given cutoff. The LGA_S scores are similar to the GDT_TS scores for closely aligned structures. LGA_S is not sensitive to out-of-sequence register errors, in the same way as the *f_m_* of a model for molecular-replacement phasing is not directly sensitive to any registration errors in the model.

A histogram of the metrics RMSD and LGA_S for the *AlphaFold*2 models across the 44 CASP crystallographic targets of interest is shown in Figs. 3 ▸(*b*) and 3 ▸(*c*). The LGA_S is skewed towards almost full sequence coverage, with an average of 87%, and the RMSD is clustered around the average RMSD of 1.27 Å.

The RMSD and LGA_S also show the superiority of the *AlphaFold*2 models over the models submitted by other groups. Table 2 ▸ shows the metrics for the *AlphaFold*2 models with the best LGA_S and the best RMSD (of the five submitted) for the 44 CASP crystallo­graphic targets of interest, compared with the best models by the same metrics overall. In only two cases (T1073 and T1085) was an *AlphaFold*2 model not the best as scored by LGA_S. In the case of T1085, the difference in the LGA_S was negligible (less than half a percent), and the *AlphaFold*2 model had a much lower RMSD (0.85 versus 1.39 Å). In the case of T1073, the differences between the models were mostly confined to a short region of the N-terminal helix that extended from the body of the globular fold. In 15 cases a non-*AlphaFold*2 model had a lower RMSD; however, this was exclusively at the expense of a lower (usually a far lower) LGA_S.

These two metrics [LGA_S and RMSD from *LGA* (4 Å)] are not ideal metrics for representing r.m.s.d. and *f_m_*. Calculation of r.m.s.d and *f_m_* is critically dependent on the alignment of the structures, and alignment should properly be based on electron density rather than coordinates, a problem that will be addressed elsewhere.

## 5. Molecular-replacement methods

The high LGA_S and low RMSD scores of the best *AlphaFold*2 models indicated that these models were good prospects for achieving phasing by molecular replacement.

Initially, the 31 targets with a one-to-one correspondence between a sequence and a CASP crystal structure, the two targets each for the two heterodimeric structures and the nine targets for PDB entry 6vr4 were used for molecular replacement (a total of 44 CASP crystallographic targets to be placed in 34 crystal structures).

If molecular replacement with the target representing the full sequence failed, and the target was one of the 11 classified as ‘multidom’, then molecular replacement was attempted with the domains.

The *AlphaFold*2 models for any given target almost exclusively superimposed with very little coordinate variability, and thus creating an ensemble structure did not indicate where poorly modelled regions could be trimmed (by divergence between models) unless exceptionally small divergence distance thresholds were used (for example 0.1 Å). Rather than using a tiny deviation threshold, trimming was performed using a threshold for the predicted error per residue supplied as part of the *AlphaFold*2 structure prediction.

The *AlphaFold*2 models (full and domain targets, un­trimmed and trimmed) were used for molecular replacement in *Phaser.voyager* (manuscript in preparation). *Phaser.voyager* uses the *phasertng* codebase (McCoy *et al.*, 2021 ▸). The initial VRMS (effective r.m.s.d.) was set to 1.2 Å and then refined for posed models. The five submitted *AlphaFold*2 models were used as an ensemble. If the target structure was available, the pose was checked to see whether it was a match for the target coordinates with *phenix.famos* (Oefner *et al.*, 2012 ▸). To confirm the solution, we used *phenix.autobuild* for the initial *R* value and *R*_free_ (Terwilliger *et al.*, 2008 ▸). Structures were considered to be solved if the correlation between σ_A_-weighted density maps computed with the *AutoBuild* model and the final structure was over 0.3. If the *R*_free_ was high, model improvement was attempted with *phenix.morph_model*. Further manual model building and refinement was not pursued.

In one case (described below) molecular replacement with *Phaser.voyager* failed, and molecular replacement was performed with *ARCIMBOLDO_LITE* for coiled coils (Caballero *et al.*, 2018 ▸).

## 6. Molecular-replacement results

Of the 34 crystal structures, 31 could be solved by molecular replacement with the *AlphaFold*2 models, two could be partially solved and one could not be solved with the *AlphaFold*2 model.

The case that could not be solved with the *AlphaFold*2 model was crystal 8, the coiled-coiled structure with PDB code 6un9, target T1048. Although not solvable with the full *AlphaFold*2 model, this structure could be solved with a generic 20-residue polyalanine helix using *ARCIMBOLDO_LITE* for coiled coils (Caballero *et al.*, 2018 ▸).

A partial solution was achieved for crystal 3, the polymerase structure with PDB code 6vr4, for which six of the nine constituent CASP targets (in two copies each) could be placed. The full structure was not designated as a CASP target. Should the whole structure, or even larger pieces, have been a target, and had an *AlphaFold*2 model been available, it is possible that such a model would have also succeeded in molecular replacement.

A partial solution was also achieved for crystal 2, the all-helical structure with PDB code 6poo. The full structure was designated as a ‘multidom’ CASP target with two domains. The second domain T1030-D1 could be placed unambiguously. The first domain T1030-D2 could be placed by molecular replacement but gave a very high final *R*_free_.

Of the 31 solved structures, 28 were solved straightforwardly with the default *Phaser.voyager* protocol (Table 3 ▸).

Details are given below for crystal 8 (no solution), crystals 2 and 3 (partial solutions) and crystals 20, 23 and 33, for which the structure solution was successful but proved to be more problematic.

### 6.1. Crystal 2 (PDB entry 6poo, target T1030)

T1030 is a helical bundle classified as ‘multidom’ with two domains. D2 could be placed unambiguously by molecular replacement, but the best pose for D1 was only able to superimpose a portion of the fragment, and the *R*_free_ after molecular replacement was greater than 0.50.

The overall C-atom r.m.s.d. of the first ranked *AlphaFold*2 model to the target for D1 was 2.8 Å over 154 residues and that for D2 was 1.2 Å over 119 residues.

The high *R*_free_ of the molecular-replacement solution can be attributed to model/target differences in the D1 bending angles and the angular disposition of the six constituent helices. Since the helices are long (residue lengths of 18, 15, 35, 15, 38 and 22) these differences result in systematic deviation of the coordinates, so that an overall r.m.s.d. does not give a complete picture of coordinate divergence.

Analysis with the *HELANAL-Plus* server (Kumar & Bansal, 2012 ▸) showed that the six *AlphaFold*2 helices were classified as ‘linear, ‘curved’, ‘linear’, ‘linear’, ‘unassigned’ and ‘curved’, while the target helices were classified as ‘curved’, ‘curved’, ‘kinked’, ‘linear’, ‘kinked’ and ‘curved’, respectively, based on the average and maximum bending angles. Analysis with *helixang* from *CCP*4 (Winn *et al.*, 2011 ▸) gave angles between the helical axes of helix 1 and helices 2–6 for the *AlphaFold*2 model of 173°, 7°, 194°, 19° and −154°, respectively, and for the target of 172°, 5°, 171°, −23° and −153°, respectively; most notable were the differences in the dispositions of helices 1 and 4 (a difference of 23°) and helices 1 and 5 (a difference of 42°).

### 6.2. Crystal 3 (PDB entry 6vr4, targets T1031, T1033, T1035, T1037 and T1039–T1043)

**Figure 4:**
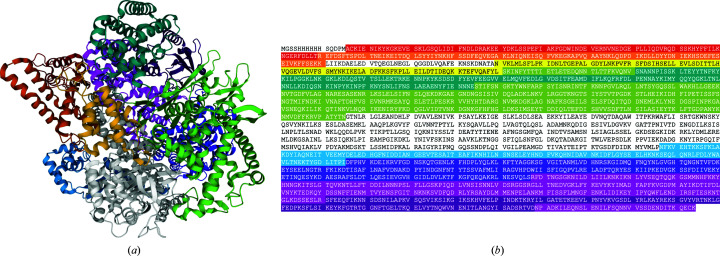
Crystal 3, PDB entry 6vr4, targets T1031 (1, red), T1033 (2, orange), T1035 (3, yellow), T1037 (4, khaki), T1039 (5, green), T1040 (6, blue), T1041 (7, purple), T1042 (8, magenta) and T1043 (9, violet). Two targets are discontinuous in the primary sequence. (*a*) Structure with targets highlighted; regions not corresponding to a target are shown in grey. The figure was created with Mol* (Sehnal *et al.*, 2021 ▸). (*b*) Sequence with targets highlighted; regions that are not highlighted were not included in targets.

For CASP14, the single polypeptide chain of the virion-packaged DNA-dependent RNA polymerase of crAss-like phage phi14:2 was divided into nine assessment domains, which we refer to here by numbering them 1–9 (Fig. 4 ▸). Eight domains were classed as FM and one was classed as FM/TBM.

There were two copies of the monomer in the asymmetric unit (PDB entry 6vr4; Drobysheva *et al.*, 2021 ▸) related by a noncrystallographic twofold. The assessment domains were used as models for molecular replacement. In total, 12 of the 18 domains could be placed, giving a 2/3 complete solution, which was insufficient for phasing the remaining fragments of the structure given the limited resolution of 3.5 Å. The partial solution was achieved by running *Phaser* from the command line. Domains were placed sequentially and the order of placement of the 12 domains was 4, 4, 7, 7, 2, 3, 2, 3, 8, 8, 5, 5. The second copies of domains 2, 3 and 8 were not placed by molecular replacement, but by applying the noncrystallographic symmetry operator to the already placed copies and performing rigid-body refinement. After the placement of the first domain 2, 40 cycles of *REFMAC* (Murshudov *et al.*, 2011 ▸) refinement were performed to improve the partial structure before continuing. This procedure was repeated after placing the second domain 3. Domains 1, 5 and 9 could not be placed; these domains had a very high r.m.s.d. to the target of over 2.5 Å (Table 4 ▸).

### 6.3. Crystal 4 (PDB entry 6n64, target T1032)

T1032 was classified as FM/TBM. There were six copies of the sequence in the asymmetric unit (Chen *et al.*, 2020 ▸) in three dimers.

The LGA_S of the model was 70%, with an RMSD of 1.7 Å. Successful molecular replacement required finding the portion of the model that was correct. The structure could be solved using two different approaches.

The first approach used *ARCIMBOLDO_SHREDDER* (Millán *et al.*, 2018 ▸), which ‘shreds’ the model into fragments defined by spheres around each C atom and uses the persistence of solutions across searches with different fragments as a way of enhancing the molecular-replacement signal. Four of the six copies of the structure in the asymmetric unit were initially found using the molecular-replacement protocol described above. These formed two dimers, each with a noncrystallographic twofold. One dimer was extracted from the partial solution and was used successfully for molecular replacement to place the final two components. Extracting oligomeric associations from a partial structure solution and using them to complete the asymmetric unit is an established protocol in molecular replacement.

The second approach used the *Phaser.voyager* pipeline after trimming the model where the predicted deviation between model and target (as generated by *AlphaFold*2) was greater than 0.7 Å. After successful molecular replacement to place all six copies using the protocol described, the complete *AlphaFold*2 model was superimposed on the fragment used for molecular replacement and refined with *phenix.morph_model* (which applies smooth distortions) to bring the *R*_free_ to 42%.

### 6.4. Crystal 8 (PDB entry 6un9, target T1048)

T1048 is a single helix and was cancelled from CASP14 on 20 October 2020 for a ‘lack of tertiary structure’. However, a model for this structure was generated by *AlphaFold*2 before the target was cancelled.

This structure is a 61-residue coiled coil. Coiled-coil structures are notoriously difficult to solve by molecular replacement because of the modulation of the data due to the helical repeats (Caballero *et al.*, 2018 ▸; Thomas *et al.*, 2015 ▸).

The structure had four copies of the target in the asymmetric unit. The overall C-alpha r.m.s.d. of the *AlphaFold*2 model to chain *A* in the target structure was 2.1 Å for 67 residues. Analysis with the *HELANAL-Plus* server (Kumar & Bansal, 2012 ▸) showed that the maximum bending angle was markedly different (7.4° versus 25.7°); the model helix was classified as ‘curved’, while the target was classified as ‘kinked’. The structure could not be solved with the *AlphaFold*2 model in *Phaser.voyager*, even with the model optimized by trimming to reduce the r.m.s.d. to the deposited structure (at the expense of a lower fraction scattering).

*ARCIMBOLDO_LITE* in coiled-coil mode was able to solve the structure using a generic 20-residue polyalanine helix. The advantage of using short generic helices for coiled-coil structures is that they are able to superimpose with multiple short sections of the coiled-coil helices with low r.m.s.d.. Structure solution required the ‘verification’ step, which is a powerful method for distinguishing the true solution from the abundance of false solutions that arise merely from helical placements that satisfy the helical modulations of the data (Caballero *et al.*, 2018 ▸).

### 6.5. Crystal 20 (target T1073)

All groups modelled the 12-residue N-terminal helix of T1073 with high r.m.s.d. to the target. This helix extends from the compact body of the fold. Structure solution was achieved by removing all sections of the *AlphaFold*2 model with a predicted error over 1 Å in a standard model-preparation protocol with *Phaser.voyager*.

The challenge in this case was with data preparation not model preparation; the model preparation was unremarkable, but we found that this case also required some additional attention be given to the crystallographic data. A number of different data sets were available in the file provided. Molecular replacement was achieved, after *phenix.xtriage* (Zwart *et al.*, 2005 ▸) analysis, with one of the data sets and with the resolution restricted to 2.8 Å.

### 6.6. Crystal 23 (target T1080)

T1080 was classified as FM/TBM. There were six copies of the target in the asymmetric unit in two trimers.

This was the only case where the five submitted *AlphaFold*2 models showed significant deviation. Model 3 differed from the consensus fold of the other four in the first 40 N-terminal residues; these 40 residues took a very different conformation in model 3. In analysis, the consensus fold of the four models was correct, and model 3 was incorrect, although the incorrect conformation could be considered as a ‘trimer swap’ error, with the chain partly following the fold of a neighbouring monomer in the trimer. The molecular-replacement model trimmed these residues and residues with a predicted deviation of more than 1.2 Å, leaving 78 of 133 residues. The molecular-replacement model was therefore 60% of the target structure. After structure solution, the full *AlphaFold*2 consensus fold was superimposed on the solution and used for refinement.

### 6.7. Crystal 33 (target T1100)

T1100 was classified as ‘multidom’ with two domains. There were four copies in the asymmetric unit in two dimers with translational noncrystallographic symmetry between the two dimers. D2 is a compact globular structure. D1 is a helical bundle structure with four helices of 52, 11, 64 and 28 residues. Within the dimer, the D1 helices formed a coiled coil.

D2 could be placed unambiguously by molecular replacement.

D1 was more difficult to place. The problem can be attributed to model/target differences in helical bending angles. In general, the helices in the model were straighter than those in the target, with average bending angles of 4.5°, 7.1°, 4.6° and 3.6° versus 8.5°, 9.3°, 8.5° and 7.8°, respectively. When the differences in bending angles were compounded over the long helices, particularly helix 1 (∼75 Å) and 3 (∼90 Å), it was not possible to simultaneously superimpose both ends of the model and the target. Molecular replacement gave several closely related poses for D1, superimposing different portions of the model and target helices.

## 7. Survey of phasing methods

To discern the impact of high-accuracy *in silico* models on crystallographic phasing methods, we undertook a survey of crystallographic phasing methods since the turn of the millennium.

We can divide crystallographic phasing strategies into four broad categories: direct methods, experimental phasing, molecular replacement and difference Fourier methods (Fourier synthesis). The use of direct-methods phasing is negligible for macromolecular crystallography, in contrast to its supreme dominance in small-molecule crystallography (Sheldrick, 2008 ▸). Within the experimental phasing category are MAD (multi-wavelength anomalous dispersion), SAD (single-wavelength anomalous dispersion) and various IR (isomorphous replacement) methods, SIR (single isomorphous replacement), SIRAS (single isomorphous replacement with anomalous scattering), MIR (multiple isomorphous replacement) and MIRAS (multiple isomorphous replacement with anomalous scattering) (for a review, see Rupp, 2010 ▸).

The PDB mostly records macromolecular crystal structures that are published in peer-reviewed journals. The PDB is a record of novel crystal forms, if not novel structures. Our analysis only included those entries where protein was a component of the crystal.

The phasing method for each PDB entry is recorded in the ‘structure determination method’ field, which should allow a survey of phasing methods; however, analysis is not straightforward for a number of reasons listed below.

(i) Although the ‘structure determination method’ field has been compulsory for submissions commenced after 29th January 2019, a significant portion of the historical entries are null. Entries recorded before 2000 were regarded as too sparse for analysis. Null entries may be biased towards particular categories of phasing.
(ii) Although the ‘structure determination method’ field has been restricted to a few text strings for submissions commenced since 29th January 2019, historically it was ‘free format’ and highly variable. For this study, all historical text entries were scanned by eye to assign each to one of the new restricted values. If the field referenced a number of methods (for example SAD with molecular replacement or SIRAS/MAD) then the most senior phasing method was allocated, with the order of precedence being MIRAS, MIR, MAD, SIRAS, SIR, SAD, molecular replacement and Fourier synthesis.
(iii) Phasing by direct methods was not included in the study because a survey of entries with the ‘structure determination method’ field *ab initio* showed that although these entries included those phased with direct methods, in the majority of cases *ab initio* referred to fragment-based approaches to molecular replacement or the use of direct methods for anomalous substructure determination. Since very few entries were categorized as *ab initio*, removing these from consideration did not significantly bias the results.
(iv) Checking a small sample of the entries in the ‘structure determination method’ field against the method recorded in the corresponding publication showed that the field was not always accurate. Inaccuracies may be biased towards particular categories of phasing.
(v) Each entry has a deposition date, a release date and a revision date, therefore dating each entry is problematic. The deposition and release dates are commonly separated by a year, but can be three years apart or even more. Revision dates are commonly very recent, as they include PDB-wide changes to PDB nomenclature. Only entries with ‘entity ID’ with value ‘1’ were considered. In order to track the evolution of structure determination methods, we considered five-year intervals and restricted PDB identifiers for each interval such that both the deposition date and the release date were within the five-year interval. Hence, our analysis sampled a subset of the PDB entries.
(vi) The understanding of the definition of different phasing methods may vary between crystallographers. For example, there is a degree of overlap between Fourier synthesis and molecular-replacement methods, as the former can be considered to be the latter but without an initial wide-radius search strategy (a search strategy employing rotation and translation functions); if the pose is outside the radius of convergence of rigid-body refinement, then (nominally) isomorphous crystals cannot be phased by difference Fourier methods and molecular replacement with a local or global search is used. Similarly, crystallographers may not distinguish between various similar experimental phasing methods (for example SAD versus SIRAS).

**Figure 5:**
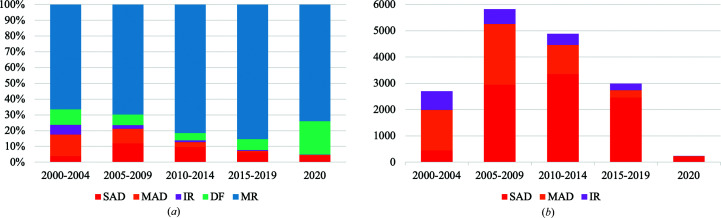
Phasing methods since 2000 as recorded in the ‘structure determination method’ field in the PDB: SAD (single-wavelength anomalous dispersion), MAD (multi-wavelength anomalous dispersion), IR (isomorphous replacement), FS (Fourier synthesis) and MR (molecular replacement). Both the deposition and release dates for the submission were within the five-year time period shown. (*a*) All methods as a percentage of PDB submissions per time period. (*b*) Experimental phasing methods as the number of PDB submissions per time period.

Despite these caveats, the trend in the change of structure determination methods over the last 20 years is clear (Fig. 5 ▸ *a*) and mirrors anecdotal experience. Molecular replacement now accounts for around 80% of phasing, increasing from around 50% of phasing in 2000, and molecular-replacement and Fourier synthesis (difference Fourier) methods combined account for 95% of phasing. It is possible that an even higher proportion of structures would have been amenable to phasing by molecular replacement had it been attempted.

Within the experimental phasing strategy, the method of experimental phasing has changed considerably, with MAD dominating in 2000 but SAD dominating today. SAD phasing, commonly using selenomethionine-substituted protein, now accounts for 82% of experimentally phased structures (Fig. 5 ▸ *b*).

Of particular note is the decline in IR methods, which were originally the backbone of macromolecular phasing. To obtain an overview of the type of structures that currently require IR to phase, we examined the structures submitted and released in 2020 in more detail (Supplementary Table S2). 13 structures met this criterion, were phased by IR methods and had publications available at the time of writing. Of these 13, we found one was actually solved by Se-SAD, one by Os-SAD, one by Pt-MAD and one by MR. Of the nine confirmed examples of IR, only two structures used multiple derivatives.

## 8. Discussion

Self-evidently, crystallography requires crystals; crystallization is a bottleneck, albeit one that has become far less constraining with advances in expression systems, fluidics, robotics and computer vision. Not only must there be crystals, but the crystals must diffract to better than 4 Å resolution to be useful for structural biology. With the data collected to the highest resolution that a crystal form (space group, unit cell, asymmetric unit contents) will allow, it is usual to regard the crystallographic data as the ‘fixed’ component of molecular-replacement phasing and to regard the model as the ‘dynamic’ component. Phasing pipelines are primarily designed for the automated exploration of many model structures prepared in different ways, with the hope that one will be accurate enough to be placed and allow model building and refinement to proceed with the single data set provided.

To some degree, the *AlphaFold*2 models upend this paradigm. The need for the extensive generation of models through different combinations of homologs in ensembles, different levels of trimming, the mining of domain databases and the use of small secondary-structure elements as models is likely to be greatly reduced as these high-accuracy models become available. In essence, the *AlphaFold*2 models distil the information from all of these methods, and more, in a single structure. The crystallographic problem may become one of finding a crystal form (for example, with fewer copies in the asymmetric unit) that is amenable to molecular replacement with the *in silico* model(s). As an example of this, successful molecular replacement with T1073 (crystal 20) was achieved after massage of the data, rather than the model.

For five of the 19 structures that were classified as ‘multidom’, we used the domains rather than the model of the whole structure for molecular replacement. In this approach, the whole structure is built up by addition as domains are placed sequentially in the asymmetric unit. It is necessary when the disposition of the domains in the target is largely determined by crystal packing or allosteric effects.

The structures that were the most challenging to solve with the *AlphaFold*2 models contained extended helices. The problem was twofold. Firstly, although helical secondary structure is very amenable to prediction, the subtle bends and kinks in the helices are more elusive, and these have long-range effects in the fit of the model to the target. Secondly, coiled coils induce modulations in the diffraction data that confound the maximum-likelihood targets in molecular replacement, a known issue and an active area of crystallo­graphic methods development.

The statistics in Table 3 ▸ show that molecular replacement with the *AlphaFold*2 models, followed by simple refinement strategies, does not give structures that are suitable for immediate submission to the PDB. Investment in manual model building, informed by a degree of biological understanding of the structures, would have been required to obtain final structures, which was beyond the scope of this study.

The use of *in silico* models for molecular replacement will also impact downstream model building and refinement. Model building and refinement can already be assisted by techniques borrowed from *ab initio* modelling (Terwilliger *et al.*, 2012 ▸). With models representing 100% of the polypeptide chain in approximately the correct conformation, model building is directed towards local minimization into electron density rather than *de novo* model building. In this study, we used *phenix.morph_model* to improve parts of the structure with an initially poor fit to the density. In regions where the electron density is weak or absent due to static disorder in the crystal, constraining the structure to the model may lower the *R* factors and improve the interpretation of the density. *In extremis*, the diffraction data may not need to be as good as they would need to be to refine, pass validation metrics and publish the structure in the absence of the model. In effect, the diffraction data need only verify the model.

There is some work to do to optimize the use of high-accuracy *in silico* models for the purposes of molecular replacement. The lack of conformational variability in the models is different from models drawn from homologs. Whereas homologs tend to vary most in the regions where they also deviate from the target structure, the *AlphaFold*2 models are very consistent (insistent) even in regions where they differ from the target structure. If taken purely at face value, this will lead to, for example, rejection of molecular-replacement solutions due to (false) packing clashes. We can also improve how we make use of the estimated error in the coordinates in model preparation. It is also likely that improvements can be made in the estimation of σ_A_ for these models, since optimization of σ_A_ estimation has been calibrated for homologs rather than *in silico* models (Hatti *et al.*, 2020 ▸).

The CASP14 crystal structures mostly represent a particular type of crystal structure: those that have a single protein sequence in the asymmetric unit and consist of one or few domains where the domain is unrelated, or poorly related, to known structures. These types of crystal structures are selected by CASP since they represent the more challenging structures for structure prediction. However, in support of structural biology, crystallography often focuses on protein complexes with peptide motifs, oligomeric associations and multi-domain structures, often with domains that already have homologous structures in the PDB. That these can already be solved by molecular replacement or Fourier synthesis in at least 95% of cases is evident in the statistics and does not diminish their scientific interest.

Our survey of phasing methods indicates that IR phasing is becoming a specialist method. Despite the undoubted power of IR to obtain spectacularly good phases, even with low resolution and poor data, there are other factors that mean that IR is avoided where possible. If using heavy metals, it requires handling highly toxic metal salts, which also bind to protein crystallographers, not just to proteins (Blundell & Johnson, 1976 ▸). Methods that incorporate noble gases such as xenon require specially designed high-pressure cells and appropriate training and support to use. We note that two of the nine structures phased by IR in our survey of 2020 were phased by SIRAS using iodine, which is a nontoxic and simple method.

Crystallographic phasing strategies have evolved continuously since 1913 (Ewald, 1962 ▸; Brooks-Bartlett & Garman, 2015 ▸), and the contribution of the high-accuracy models is continuing this evolution. It is already enabling crystallo­graphers to concentrate their efforts even more keenly on the structural biology by making crystallographic phasing even more straightforward. We should look forward to the biological insights that this will bring.

## 9. Related literature

The following references are cited in the supporting information for this article: Bahat *et al.* (2020 ▸), Eckenroth *et al.* (2021 ▸), Filipčík *et al.* (2020 ▸), Gandini *et al.* (2020 ▸), Garcia-Doval *et al.* (2020 ▸), He *et al.* (2020 ▸), Jäger *et al.* (2020 ▸), Jensen *et al.* (2020 ▸), Micevski *et al.* (2020 ▸), Nie *et al.* (2021 ▸), Sakurai *et al.* (2020 ▸), Zhu *et al.* (2020 ▸) and Zimanyi *et al.* (2020 ▸).

## Supplementary Material

Supplementary figure and tables. DOI: 10.1107/S2059798321012122/qg5003sup1.pdf

## Figures and Tables

**Table 1:** The 34 crystal structures included in CASP14 and the targets associated with each crystal structure

Crystal No.	Target No.	CASP target	Residues	CASP domain	Residues	‘Multidom’ domains(s)	Residues
1	1	T1024	408			T1024-D1	193
						T1024-D2	204
2	2	T1030	273	T1030-D0	(273)	T1030-D1	154
						T1030-D2	119
3	3	T1031	95	T1031-D1	(95)		
	4	T1033	100	T1033-D1	(100)		
	5	T1035	102	T1035-D1	(102)		
	6	T1037	404	T1037-D1	(404)		
	7	T1039	161	T1039-D1	(161)		
	8	T1040	130	T1040-D1	(130)		
	9	T1041	242	T1041-D1	(242)		
	10	T1042	289	T1042-D1	276		
	11	T1043	148	T1043-D1	(148)		
4	12	T1032	284	T1032-D1	169		
5	13	T1034	156	T1034-D1	(156)		
6	14	T1038	199	T1038-D0	190	T1038-D1	114
						T1038-D2	76
7	15	T1046s1	216	T1046s1-D1	72		
	16	T1046s2	216	T1046s2-D1	141		
8	17	T1048†					
9	18	T1049	141	T1049-D1	134		
10	19	T1050	779			T1050-D1	321
						T1050-D2	316
						T1050-D3	128
11	20	T1052	832	T1052-D0	(832)		
12	21	T1053	580	T1053-D0	576	T1053-D1	405
						T1053-D2	171
13	22	T1054	190	T1054-D1	143		
14	23	T1056	186	T1056-D1	169		
15	24	T1058	382	T1058-D0	(382)	T1058-D1	221
						T1058-D2	161
16	25	T1064	106	T1064-D1	92		
17	26	T1065s1	225	T1065s1-D1	11		
	27	T1065s2	225	T1065s2-D1	98		
18	28	T1067	220	T1067-D1	(221)		
19	29	T1070	335			T1070-D1	76
						T1070-D2	101
						T1070-D3	76
						T1070-D4	68
20	30	T1073	58	T1073-D1	(59)		
21	31	T1074	131	T1074-D1	(132)		
22	32	T1079	483	T1079-D1	451		
23	33	T1080	137	T1080-D1	133		
24	34	T1082	97	T1082-D1	75		
25	35	T1083	196	T1083-D1	92		
26	36	T1084	146	T1084-D1	71		
27	37	T1085	588	T1085-D0	406	T1085-D1	167
						T1085-D2	182
						T1085-D3	57
28	38	T1086	408	T1086-D0	381	T1086-D1	193
						T1086-D2	188
29	39	T1087	186	T1087-D1	93		
30	40	T1089	404	T1089-D1	377		
31	41	T1090	193	T1090-D1	191		
32	42	T1091	863			T1091-D1	139
						T1091-D2	107
						T1091-D3	106
						T1091-D4	112
33	43	T1100	338			T1100-D1	171
						T1100-D2	166
34	44	T1101	318	T1101-D0	307	T1101-D1	83
						T1101-D2	224

† Cancelled.

**Table 2:** Best models for the targets for the 34 crystal structures included in CASP14 The model is given as (CASP group No.)_(ranked model No.). In brackets are the LGA_S and RMSD. Group Nos.: 427, AlphaFold2; 013, FEIG-S; 029, Venclovas; 071, Kiharalab; 080, FOLDYNE; 081, MUFOLD; 129, Zhang; 132, PBuild; 140, Yang-Server; 217, CAO-QA1; 259, AWSEM-CHEN; 288, DATE; 334, FEIG-R3; 337, CATHER; 392, trfold; 403, BAKER-experimental; 473, BAKER; 480, FEIG-R2.

Crystal No.	Target(s) in ASU	Best *AlphaFold*2 model by LGA_S	Best model overall by LGA_S	Best *AlphaFold*2 model by RMSD	Best model overall by RMSD
1	T1024	427_3 [87.5, 1.83]	427_3	427_1 [58.8, 1.60]	427_1
2	T1030	427_2 [62.0, 1.82]	427_2	427_2	013_2 [39.2, 1.27]
3	T1031	427_2 [94.0, 1.12]	427_2	427_4 [93.7, 0.98]	427_4
	T1033	427_1 [93.3, 1.39]	427_1	427_3 [92.5, 1.36]	259_4 [39.3, 1.29]
	T1035	427_5 [99.0, 0.81]	427_5	427_5	427_5
	T1037	427_4 [95.4, 1.12]	427_4	427_5 [93.7, 1.11]	427_5
	T1039	427_1 [86.3, 1.61]	427_1	427_1	071_1 [33.5, 1.17]
	T1040	427_1 [77.5, 1.95]	427_1	427_2 [76.3, 1.90]	140_1 [16.4, 1.31]
	T1041	427_1 [94.7, 1.21]	427_1	427_1	427_1
	T1042	427_3 [93.8, 1.22]	427_3	427_5 [93.4, 1.21]	427_5
	T1043	427_3 [90.2, 1.42]	427_3	427_1 [90.0, 1.41]	427_1
4	T1032	427_3 [71.1, 1.67]	427_3	427_1 [70.1, 1.65]	427_1
5	T1034	427_1 [96.9, 1.00]	427_1	427_2 [95.7, 0.87]	427_2
6	T1038	427_2 [91.9, 1.17]	427_2	427_2	427_2
7	T1046s1	427_4 [98.1, 0.68]	427_4	427_1 [98.1, 0.64]	427_1
	T1046s2	427_1 [98.9, 0.69]	427_1	427_1	427_1
8	T1048†				
9	T1049	427_1 [95.3, 0.82]	427_1	427_1	427_1
10	T1050	427_1 [93.3, 1.26]	427_1	427_1	427_1
11	T1052	427_4 [63.4, 1.17]	427_4	427_5 [62.9, 1.14]	337_5 [45.5, 1.13]
12	T1053	427_3 [96.9, 0.98]	427_3	427_3	427_3
13	T1054	427_3 [93.7, 0.84]	427_3	427_2 [93.0, 0.81]	029_1 [49.7, 0.76]
14	T1056	427_2 [99.3, 0.66]	427_2	427_2	427_2
15	T1058	427_3 [93.7, 1.25]	427_3	427_3	427_3
16	T1064	427_1 [92.6, 1.34]	427_1	427_2 [91.0, 1.31]	427_2
17	T1065s1	427_2 [98.4, 0.91]	427_2	427_4 [97.8, 0.85]	427_4
	T1065s2	427_1 [99.5, 0.60]	427_1	427_1	427_1
18	T1067	427_3 [92.9, 0.86]	427_3	427_3	427_3
19	T1070	427_5 [45.0, 1.69]	427_3	427_3 [41.2, 1.52]	334_1 [30.4, 1.22]
20	T1073	427_3 [86.7, 1.76]	288_4 [95.2, 1.47]	427_5 [85.5, 1.41]	217_3 [83.8, 0.97]
21	T1074	427_2 [93.7, 1.15]	427_2	427_4 [92.6, 1.06]	427_4
22	T1079	427_4 [96.7, 1.05]	427_4	427_2 [96.7, 1.03]	427_2
23	T1080	427_4 [91.6, 1.37]	427_4	427_4	427_4
24	T1082	427_1 [97.9, 0.88]	427_1	427_2 [97.3, 0.88]	427_2
25	T1083	427_4 [91.9, 1.09]	427_4	427_4	392_2 [88.7, 0.96]
26	T1084	427_5 [94.6, 0.85]	129_3 [95.1, 1.39]	427_4 [94.0, 0.77]	480_4 [92.6, 0.60]
27	T1085	427_1 [88.2, 1.86]	427_1	427_1	473_4 [32.5, 1.45]
28	T1086	427_1 [89.6, 1.78]	427_1	427_4 [88.7, 1.64]	080_1 [53.0, 1.60]
29	T1087	427_3 [97.0, 0.63]	427_3	427_2 [96.8, 0.57]	081_3 [40.3, 0.38]
30	T1089	427_2 [99.0, 0.71]	427_2	427_2	427_2
31	T1090	427_3 [95.4, 1.16]	427_3	427_1 [92.1, 1.04]	427_1
32	T1091	427_2 [79.8, 2.00]	427_2	427_5 [77.0, 1.96]	403_2 [27.3, 1.47]
33	T1100	427_2 [90.3, 1.68]	427_2	427_4 [55.9, 1.06]	132_2 [18.2, 0.81]
34	T1101	427_4 [92.0, 1.17]	427_4	427_3 [91.9, 1.15]	427_3

† Cancelled

**Table 3:** Summary of phasing of the 34 crystal structures of interest in CASP14 with *AlphaFold*2 models Crystals and targets listed in bold are discussed in the text.

Crystal	Target	PDB code	*d*_min_ (Å)	No. of reflections	*Z* †	Domains	Filter‡	TFZ§	*R*_free_	*R*_free_ after morphing
1	T1024	6t1z	2.9	12686	1	1	No	24.7	0.52	0.42
**2** ¶	**T1030**	**6poo**	**3.0**	**7525**	**1**	**2**	**No**	**20.4**	**0.53**	
**3** ¶	**T1031**	6vr4	3.5	92907	2					
	**T1033**									
	**T1035**									
	**T1037**									
	**T1039**									
	**T1040**									
	**T1041**									
	**T1042**									
	**T1043**									
**4**	**T1032**	**6n64**	**3.3**	**27936**	**6**	**1**	**0.7**	**16.1**	**0.47**	**0.44**
5	T1034	6tmm	2.1	47702	4	1	No	31.9	0.45	
6	T1038	6ya2	2.5	20426	3	1	No	24.3	0.35	
7	T1046s1	6px4	1.7	69112	2	2	No	31.8	0.35	
	T1046s2									
**8** ††	**T1048**	**6un9**	**2.8**	**19203**	**4**					
9	T1049	6y4f	1.8	12228	1	1	No	23.7	0.34	
10	T1050		2.7	97731	3	1	No	39.0	0.30	
11	T1052		2.0	88914	2	1	No	48.6	0.43	
12	T1053	7m7a	3.2	49627	4	1	No	51.0	0.35	
13	T1054	6v4v	1.7	25547	1	1	No	33.2	0.34	
14	T1056	6yj1	2.3	17863	2	1	No	19.7	0.37	
15	T1058	7abw	3.1	20228	2	1	No	25.5	0.44	
16	T1064	7jtl	2.0	16787	2	1	No	19.1	0.41	
17	T1065s1	7m5f	1.6	35695	1	2	No	48.7	0.22	
	T1065s2									
18	T1067		1.4	51025	1	1	No	57.2	0.26	
19	T1070		2.5	25412	1	1	No	6.8	0.49	0.42
**20**	**T1073**		**1.9**	**27326**	**4**	**1**	**1**	**24.1**	**0.39**	
21	T1074	7oc9	1.5	25800	1	1	No	21.1	0.29	
22	T1079		3.2	47985	4	1	No	33.6	0.38	
**23**	**T1080**		**1.7**	**100570**	**6**	**1**	**No**	**20.9**	**0.39**	
24	T1082	6x6o	1.1	97672	2	1	No	33.6	0.44	0.31
25	T1083		1.3	81236	4	1	0.6	30.0	0.48	0.35
26	T1084		1.9	23901	3	1	No	32.1	0.38	
27	T1085		2.5	10758	1	3	No	22.0	0.40	
28	T1086		2.3	21887	1	1	No	18.7	0.41	
29	T1087		1.4	69617	4	1	No	43.2	0.25	
30	T1089		2.2	55192	2	1	No	63.5	0.29	
31	T1090	7k7w	1.8	22947	1	1	No	27.2	0.29	
32	T1091		2.2	62789	1	4	No	22.5	0.44	
**33**	**T1100**		**3.1**	**36829**	**4**	**1**	**No**	**31.1**	**0.48**	**0.45**
34	T1101		1.4	58030	1	1	No	16.2	0.34	

† Number of copies in the asymmetric unit.

‡ Threshold for predicted RMSD below which residues were pruned from the model.

§ Translation-function *Z*-score, a measure of significance of the MR solution.

¶ Partial solution.

†† No solution.

**Table 4:** Phasing of crystal 3 (PDB entry 6vr4) with *AlphaFold*2 models For CASP14, the single polypeptide chain of the target sequence was divided into nine assessment domains. There were two copies of the target sequence in the crystallographic asymmetric unit. Two copies each of six targets were found by molecular replacement and given chain identifiers *A*–*L* in order of placement. Targets T1031, T1040 and T1043 could not be placed.

	Model	*Z*	Type	Residues	R.m.s.d. (Å)	Chain
1	T1031TS427_1-D1	2	FM	95	2.91	
2	T1033TS427_1-D1	2	FM	100	1.58	*EG*
3	T1035TS427_1-D1	2	FM/TBM	102	0.81	*FH*
4	T1037TS427_1-D1	2	FM	404	1.25	*AB*
5	T1039TS427_1-D1	2	FM	161	2.50	*KL*
6	T1040TS427_1-D1	2	FM	130	2.76	
7	T1041TS427_1-D1	2	FM	242	1.70	*CD*
8	T1042TS427_1-D1	2	FM	276	1.79	*IJ*
9	T1043TS427_1-D1	2	FM	148	2.46	
