# Supplementary material for: Implications of AlphaFold2 for crystallographic phasing by molecular replacement
Source: Acta Crystallogr D Struct Biol. 2022 Jan 1;78(Pt 1):1–13. doi: 10.1107/S2059798321012122 (PMC8725160; doi:10.1107/S2059798321012122)
Supplement: Supplementary file 1 [file d-78-00001-sup1.pdf]

STRUCTURAL BIOLOGY

Volume 78 (2022)

Supporting information for article:

Implications of *AlphaFold*2 for crystallographic phasing by molecular replacement

Airlie J. McCoy, Massimo D. Sammito and Randy J. Read

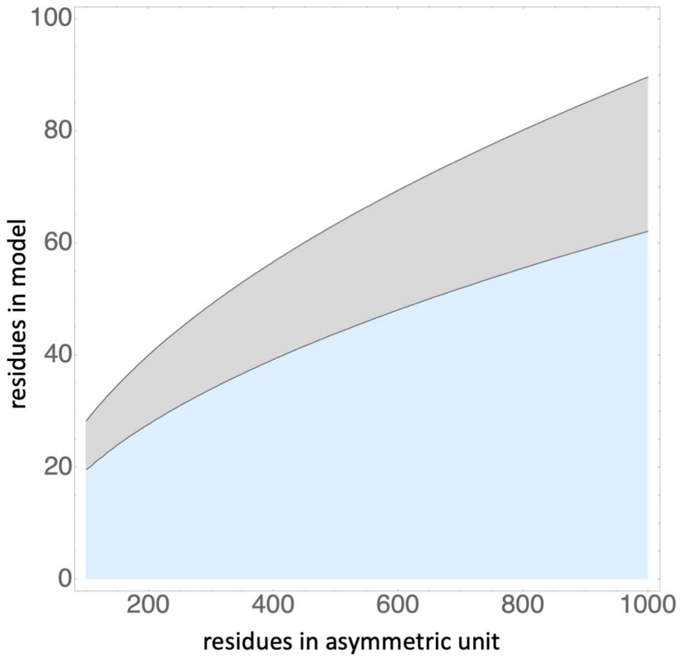

Figure S1 Expected LLG for an accurate model (0.4 Å RMS) given data to 2.2 Å resolution. The eLLG is shown as a function of asymmetric unit content (measured in amino acid residues) and the size of the model (also in residues). In computing the number of reflections expected, a solvent content of 50% was assumed. The lower contour bordering the blue area represents an eLLG of 60 and the higher contour bordering the grey area an eLLG of 125. LLG values above 60 generally indicate success when placing a first component, whereas the value of 125 is the default target eLLG used by Phaser to make strategic choices about resolution and model size. This plot indicates, for instance, that attempts to place an accurate model comprising as little as 25-40 residues should be successful when the asymmetric unit contains 200 residues, whereas a model comprising 50-80 residues would be needed for an asymmetric unit of 800 residues.

Table S1 Crystallographic targets of interest showing organism of origin and prediction classification.

Free modelling (FM), template-based modelling (TBM) and borderline between free modelling and template-based modelling (FM/TBM).

Crystal	Organism	Target	Category
1 6t1z	Lactococcus cremoris	T1024	TBM-easy
2 **6poo**	**Streptococcus agalactiae**	T1030	MultiDom
		T1030-D1	TBM-hard
		T1030-D2	TBM-hard
3 6vr4	*Cellulophaga baltica* crAss-like phage phi14:2	T1031	FM
		T1033	FM
		T1035	FM/TBM
		T1037	FM
		T1039	FM
		T1040	FM
		T1041	FM
		T1042	FM
		T1043	FM
4 6n64	Mus musculus (SMCHD1)	T1032	TBM-hard
5 6tmm	**Tetrahymena thermophila**	T1034	TBM-easy
6 6ya2	Tomato spotted wilt tospovirus	T1038	Multidom
		T1038-D1	FM
		T1038-D2	FM/TBM
7 6px4	**Escherichia phage ECML-134**	T1046s1	TBM-hard
		T1046s2	
9 6y4f	Proteus mirabilis	T1049	FM
10	Bacteroides Ovatus	T1050	TBM-easy
11	Salmonella enterica	T1052	TBM-easy
12	Legionella pneumophila	T1053	FM/TBM
13	Acinetobacter baumannii	T1054	TBM-hard
14	Staphylococcal phage	T1056	TBM-hard
15	Pseudomonas aeruginosa	T1058	FM/TBM
16 7jtl	SARS-CoV2	T1064	FM
17	Serratia marcescens	T1065s1	FM/TBM
		T1065s2	
18	Bdellovibrio bacteriovorus	T1067	TBM-hard
19	Escherichia virus CBA120	T1070	FM
		T1070-D1	TBM-easy
		T1070-D2	TBM-hard
		T1070-D3	TBM-easy
		T1074-T4	
20	Bdellovibrio bacteriovorus	T1073	TBM-easy
21	Bdellovibrio bacteriovorus	T1074	FM
22	Bdellovibrio bacteriovorus	T1079	TBM-hard
23	Bdellovibrio bacteriovorus	T1080	FM/TBM
24 6x6o	T4 phage	T1082	FM/TBM
25	Nitrosococcus oceani	T1083	TBM-hard
26	Meiothermus silvanus	T1084	TBM-hard
27	Bdellovibrio bacteriovorus	T1085	Multidom
		T1085-D1	TBM-hard
		T1085-D2	FM/TBM
		T1085-D3	TBM-hard
28	Bdellovibrio bacteriovorus	T1086	Multidom
		T1086-D1	TBM-easy
		T1086-D2	TBM-hard
29	Methylobacter tundripaludum	T1087	TBM-hard
30	Bacteroides finegoldii	T1089	TBM-easy
31 7k7w	Schizosaccharomyces pombe	T1090	FM
32	Streptococcus oralis	T1091	TBM-easy
33	Archaeoglobus fulgidus	T1100	TBM-hard
34	Bilateria	T1101	Multidom
		T1101-D1	TBM-easy
		T1101-D2	TBM-easy

Table S2 Crystals phased by isomorphous replacement, that were reported to the PDB, contained protein and had both a deposition date and release date in 2020.

PDB phasing method as recorded in the ‘structure determination method’ field.

	PDBID	resolution	PDB Phasing Method	Publication Phasing Method	Details	Publication
1	6TUG	2.4	SIRAS	SIRAS	native + Hg	(Garcia-Doval *et al.*, 2020)
2	6VJI	2.5	MIRAS	MAD/MIRAS	Se-MAD + Pt + Au + native	(Eckenroth *et al.*, 2021)
3	6VW9	1.8	SIRAS	SIRAS	native + I	(Zimanyi *et al.*, 2020)
4	6W8R	2.8	MIRAS	SIRAS	native + Se	(Zhu *et al.*, 2020)
5	6WA6	2.8	MIRAS	MIRAS	Au + Pt + U + native	(Jensen *et al.*, 2020)
6	6WHB	1.9	SIRAS	SIRAS	native + I	(Filipčík *et al.*, 2020)
7	6WN2	1.8	MIR	MR		(He *et al.*, 2020)
8	6Y7F	2.1	SIRAS	SIRAS	native + Hg	(Nie *et al.*, 2021)
9	6Y9L	4.1	MIRAS	SAD	Os-SAD	(Bahat *et al.*, 2020)
10	6YV7	2.7	SIRAS	SIRAS	native + W	(Gandini *et al.*, 2020)
11	6Z4E	2.0	MIR	MAD	Pt-MAD	(Micevski *et al.*, 2020)
12	7AED	1.8	SIRAS	SAD	Se-SAD	(Jäger *et al.*, 2020)
13	7BQI	1.3	SIRAS	SIRAS	native + Hg	(Sakurai *et al.*, 2020)
